# Predicting continuous amyloid PET values with CSF tau phosphorylation occupancies

**DOI:** 10.1002/alz.14132

**Published:** 2024-07-23

**Authors:** Julie K. Wisch, Brian A. Gordon, Nicolas R. Barthélemy, Kanta Horie, Rachel L. Henson, Yingxin He, Shaney Flores, Tammie L. S. Benzinger, John C. Morris, Randall J. Bateman, Beau M. Ances, Suzanne E. Schindler

**Affiliations:** ^1^ Department of Neurology Washington University in St. Louis St. Louis Missouri USA; ^2^ Department of Radiology Washington University in St. Louis St. Louis Missouri USA; ^3^ Knight Alzheimer Disease Research Center Washington University School of Medicine St Louis Missouri USA; ^4^ SILQ Center for Neurodegenerative Biology St. Louis Missouri USA; ^5^ Hope Center Washington University in Saint Louis St. Louis Missouri USA

**Keywords:** biomarker concordance, CSF tau occupancy, machine learning, novel biomarkers, PET

## Abstract

**INTRODUCTION:**

Cerebrospinal fluid (CSF) tau phosphorylation at multiple sites is associated with cortical amyloid and other pathologic changes in Alzheimer's disease. These relationships can be non‐linear. We used an artificial neural network to assess the ability of 10 different CSF tau phosphorylation sites to predict continuous amyloid positron emission tomography (PET) values.

**METHODS:**

CSF tau phosphorylation occupancies at 10 sites (including pT181/T181, pT217/T217, pT231/T231 and pT205/T205) were measured by mass spectrometry in 346 individuals (57 cognitively impaired, 289 cognitively unimpaired). We generated synthetic amyloid PET scans using biomarkers and evaluated their performance.

**RESULTS:**

Concentration of CSF pT217/T217 had low predictive error (average error: 13%), but also a low predictive range (ceiling 63 Centiloids). CSF pT231/T231 has slightly higher error (average error: 19%) but predicted through a greater range (87 Centiloids).

**DISCUSSION:**

Tradeoffs exist in biomarker selection. Some phosphorylation sites offer greater concordance with amyloid PET at lower levels, while others perform better over a greater range.

**Highlights:**

- Novel pTau isoforms can predict cortical amyloid burden.
- pT217/T217 accurately predicts cortical amyloid burden in low‐amyloid individuals.
- Traditional CSF biomarkers correspond with higher levels of amyloid.

## 1 INTRODUCTION

A key line of inquiry in Alzheimer's disease (AD) research has focused on developing fluid biomarkers predictive of neuropathology. Amyloid plaques and tau neurofibrillary tangles, hallmarks of AD, are visualized in vivo with positron emission tomography (PET).^1^, ^2^ PET has excellent concordance with plaque burden at autopsy^3^, ^4^, ^5^ but requires specialized equipment and is more expensive than cerebrospinal fluid (CSF). CSF assays have been developed that measure Aβ42, Aβ40, tau phosphorylated at site 181 (p‐tau181), and total tau (t‐tau).^6^, ^7^ CSF biomarker ratios, including Aβ42 (p‐tau181/Aβ42, t‐tau/Aβ42, and Aβ42/Aβ40), have good, but not perfect, agreement with amyloid PET.^5^, ^8^, ^9^, ^10^, ^11^ Although collection of CSF requires a lumbar puncture (LP), which is perceived by some individuals as invasive and is associated with mild potential risks, CSF testing for AD biomarker assessment is used by many memory specialty clinics.^12^, ^13^

We previously evaluated the continuous relationship between CSF Aβ42/Aβ40 and amyloid PET.^14^ We generated forecasts of regional amyloid accumulation with a mean average percent error (MAPE) of approximately 14%.^14^ CSF Aβ42/Aβ40 declines and then reaches a plateau as amyloid burden increases.^6^ We identified the maximum amyloid burden at which lower CSF Aβ42/Aβ40 was associated with higher amyloid burden (2.60 standardized uptake value ratio [SUVR], 69.5 Centiloids).^14^ This plateau demonstrates that at high levels of amyloid burden, variance in CSF Aβ42/Aβ40 no longer reflects continued pathological accumulation.

p‐tau181 has commonly been used as the CSF measure of tau. However, more than 40 tau sites are phosphorylated in brain,^15^ and different tau CSF phosphorylation sites change continuously over the course of AD, with some sites more strongly associated with amyloid PET and others more strongly associated with tau PET.^16^, ^17^ Recent work suggests the phosphorylation occupancy (the ratio of phosphorylated to non‐phosphorylated tau) at threonine 217 and 231 (pT217/T217 and pT231/T231) has stronger associations with amyloid PET than Aβ42/Aβ40 or p‐tau181.^7^, ^17^, ^18^, ^19^, ^20^ Phosphorylation occupancies at threonine 111 and 153 (pT111/T111, pT153/T153) as well as serine 208 (pS208/S208) have also been found to have strong associations with amyloid PET specifically in amyloid‐positive individuals.^21^ The phosphorylation occupancy at threonine 205 (pT205/T205) is more associated with brain tau aggregation.^7^, ^17^, ^21^, ^22^

Many studies evaluating the utility of these phosphorylation sites have examined the relationship between p‐tau concentrations and dichotomous amyloid PET status.^7^, ^9^, ^11^, ^20^ When evaluating the performance of biomarkers in the context of imbalanced data (eg, cohorts primarily composed of amyloid‐negative individuals), Area Under the Curve (AUC) can be misleading as it treats true positive and false positive rates equally.^23^ As an example, a model designed to predict amyloid positivity in a cohort of 80 amyloid‐negative individuals and 20 amyloid‐positive individuals could achieve an AUC of 0.80 by predicting that all individuals are amyloid negative. While appearing to perform well, this model is devoid of information about an individual's amyloid pathology. When the case of interest is the rarer case, as is often true in research pertaining to preclinical AD, it is important to consider alternative approaches to understanding the strength and consistency of relationships between fluid biomarkers and amyloid PET.

In this study, we applied a machine learning approach to evaluate the non‐linear relationship between CSF tau phosphorylation at 10 sites and continuous amyloid PET burden. We compared the performance of the tau phosphorylation occupancies as measured by mass spectrometry (MS) to CSF Aβ42/Aβ40 and p‐tau181/Aβ42 as measured by clinically used immunoassays. We produce both aggregate error measurements like MAPE and evaluations of error across the full range of predictive values.

## 2 METHODS

### 2.1 Participants

Older adults enrolled in studies of memory and aging at the Charles F. and Joanne Knight Alzheimer Disease Research Center (Knight ADRC) at Washington University in St. Louis who underwent CSF collection within 2 years of an amyloid PET scan were included. Written informed consent was obtained from all participants. Washington University's Institutional Review Board approved all procedures. Participants completed annual clinic visits including assessment of Clinical Dementia Rating (CDR).^24^ *APOE* genotyping was performed using either an Illumina 610 or OmniExpress chip.^25^

### 2.2 Cerebrospinal fluid biomarkers

CSF samples were collected in the morning following overnight fasting.^6^, ^11^ Concentrations of CSF Aβ40, Aβ42, and p‐tau181 were measured by chemiluminescent enzyme immunoassay using a fully automated platform (LUMIPULSE G1200, Fujirebio, Malvern, PA, USA). An immunoprecipitation‐MS (IPMS) assay was used to measure CSF tau phosphorylation occupancies (%phosphorylated to non‐phosphorylated tau) at T111, T153, T175, T181, S199, S202, T205, S208, T217, and T231.^21^, ^26^

### 2.3 Positron emission tomography imaging

Amyloid PET scans with ^11^C‐Pittsburgh Compound B (PiB) were obtained, and images were processed using the PET Unified Pipeline (PUP, https://github.com/ysu001/PUP),^27^, ^28^ consistent with previous descriptions of PET image processing.^14^ PET images were co‐registered with T1‐weighted scans that had been segmented with FreeSurfer 5.3 using the Desikan–Killiany atlas. The cerebellar cortex was used as the reference region. An amyloid PET summary value was calculated from the arithmetic mean of SUVR values for the following bilateral regions: precuneus, superior frontal and rostral middle frontal regions, lateral orbitofrontal and medial orbitofrontal regions, and superior temporal and middle temporal regions.^28^ Individuals were considered amyloid positive if their cortical amyloid burden was greater than 1.42 SUVR (16.4 Centiloids).^28^ SUVRs were converted to Centiloids (relying on the cerebellar cortex reference region as calculated within the Global Alzheimer's Association Information Network dataset) in order to increase comparability with other published studies.^29^

### 2.4 Statistical analysis

Spearman correlations were used to evaluate the strength of the relationship between each individual CSF biomarker and cortical amyloid burden.

### 2.5 Model development and architecture

We constructed feedforward artificial neural networks (ANNs) in an identical manner to our previously published approach.^14^ ANNs enable the prediction of multiple output values (in this case, regional PET values) from as little as a single input. The training of this type of model relies on the underlying covariance structure of the multiple outputs. The input to the ANNs was each CSF measure individually, and the output of the models corresponded to 37 bilateral regional PET‐PiB SUVR values defined by Desikan–Killany parcellation. All models were trained on 80% of the data, with 20% held out for testing.

The ANNs were constructed with an input layer, three hidden layers with rectified linear unit activation functions, and an output layer with a linear activation function. We used dropout between layers to reduce the risk of overfitting.^30^ The ANNs were trained using adaptive moment estimation (ADAM) optimization. We terminated training after 100 epochs. Hyperparameter optimization via coarse grid search was applied as previously described.^14^ Training optimization was based on minimizing the mean squared error; however, we present MAPE to facilitate interpretation of results. Confidence intervals (CIs) were constructed by applying bootstrap resampling to 80% of the training set (64% of the total dataset) and applying hyperparameter tuning to a subset of potential parameters.

**RESEARCH IN CONTEXT**

1. **Systematic review**: Cerebrospinal fluid (CSF) biomarkers of Alzheimer's disease (AD) have continuous non‐linear relationships with amyloid positron emission tomography (PET) burden. Although amyloid PET status (positive/negative) is frequently used in analyses, dichotomization reduces the information available from this measure. We previously characterized the continuous relationship between CSF Aβ42/Aβ40 and amyloid PET. Recent advances in mass spectrometry have allowed for the measurement of phosphorylation occupancies at multiple tau sites and demonstrated that several occupancies have high correspondence with amyloid pathology.
2. **Interpretation**: CSF pT217/T217 (%) was the best predictor of amyloid PET, but it does not consistently increase (especially after 63 Centiloids). CSF Aβ42/Aβ40, p‐tau181/Aβ42, and pT231/T231 had slightly lower accuracy for predicting amyloid PET but had a greater range (through 87 Centiloids).
3. **Future directions**: Validation of this continuous modeling approach is necessary in additional cohorts. Expansion of this technique to plasma biomarkers and other PET tracers should be considered.

### 2.6 Evaluation of model performance

The neural network models were used to generate regional SUVR predictions, which were evaluated at both the regional and global levels. The MAPE was calculated by taking the average of the error associated with each individual forecast as compared to the actual value. We also assessed the performance of the models at a range of mean cortical values. We calculated the MAPE across a 25‐unit sliding window, applying a 1000‐iteration bootstrap, as previously described.^14^

### 2.7 Inference from developed models

We applied asymptotic regression to quantify the upper limit of each model that forecasted mean cortical SUVR as previously described^14^ using a non‐linear least‐squares model.^31^ CIs were constructed based on standard error estimates from the non‐linear least‐squares model.

## 3 RESULTS

The cohort included 346 participants (μ_age_ = 70.2 years, ơ^2^_age_ = 8.9 years). Most individuals were White (89.6%), 51.4% were female, 37.3% carried an *APOE* ε4 allele, and 36.7% were amyloid positive by PET. The majority of participants were cognitively unimpaired (83.5% were rated CDR 0), while 13.9% had very mild dementia (CDR = 0.5) and 2.6% had mild dementia (CDR 1). Most participants (59.2%) were very clearly amyloid negative with an amyloid burden less than 10 Centiloids, 11% had an intermediate level of amyloid burden (10 to 30 Centiloids), and 29.8% had significant amyloid burden (greater than 30 Centiloids) (Table 1).

**TABLE 1:** Participant characteristics.

	Overall
*N*	346
Age at lumbar puncture (mean [SD])	70.2 (8.9)
Sex = Female (%)	178 (51.4)
Race = White (%)	310 (89.6)
Clinical Dementia Rating (%)	
0	289 (83.5)
0.5	48 (13.9)
1	9 (2.6)
*APOE* ε4 carrier (%)	128 (37.3)
Years between LP and PET sessions (mean [SD])	0.36 (0.47)
Amyloid status = Amyloid PET positive (%)	127 (36.7)
Mean cortical amyloid burden (SUVR) (mean [SD])	1.62 (0.87)

Abbreviations: LP, lumbar puncture; PET, positron emission tomography; SUVR, standardized uptake value ratio.

**FIGURE 1:**
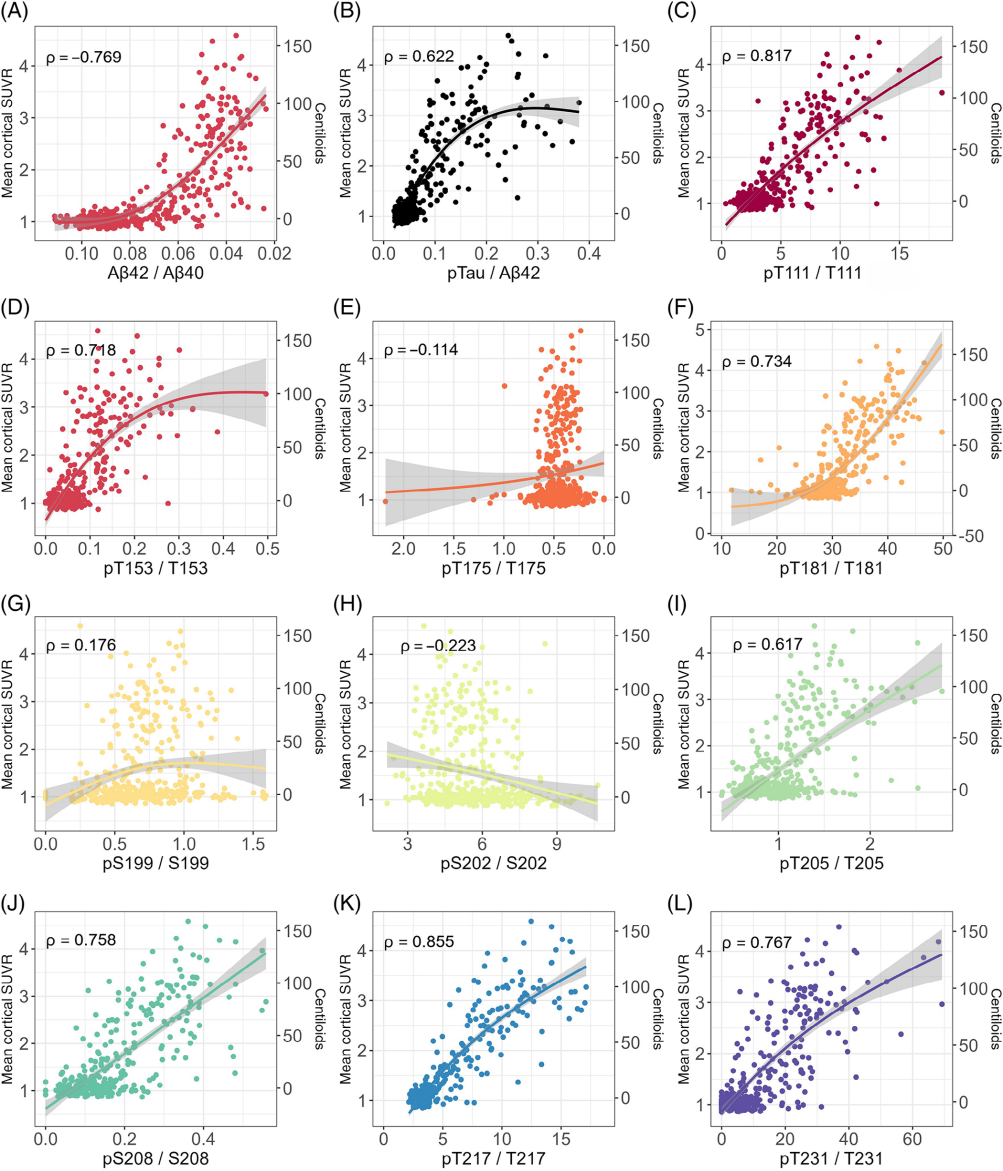
Relationship between individual fluid biomarkers and cortical amyloid burden.

Mean cortical amyloid burden by PET was significantly correlated with CSF Aβ42/Aβ40 and p‐tau181/Aβ42 as measured by Lumipulse (Figure 1A–B) as well as each of the 10 CSF tau phosphorylation occupancies (Figure 1C–L). The strongest Spearman correlations were between mean cortical amyloid burden and pT217/T217 (*ρ* = 0.855, 95% CI: 0.827, 0.879), pT111/T111 (*ρ* = 0.817, 95% CI: 0.783, 0.847), and pS208/S208 (*ρ* = 0.758, 95% CI: 0.712, 0.798). Lumipulse CSF Aβ42/Aβ40 (*ρ* = −0.769, 95% CI: −0.806, −0.727) and p‐tau181/Aβ42 (*ρ* = 0.622, 95% CI: 0.559, 0.677) were also strongly correlated with mean cortical amyloid burden.

Next, we used ANNs to predict continuous amyloid PET values using each of the 10 tau phosphorylation occupancies as well as Lumipulse CSF Aβ42/Aβ40 and p‐tau181/Aβ42 (Figure S1). The CSF pT217/T217 model had the lowest error in predicting amyloid PET burden compared to the other tau phosphorylation occupancies (MAPE = 13.0%), followed by CSF pT231/T231 (MAPE = 19.0%), and CSF pT111/T111 (19.9%). Both Lumipulse‐derived measures had error rates similar to CSF pT231/T231; CSF p‐tau181/Aβ42 had a MAPE of 18.1% and CSF Aβ42/Aβ40 had a MAPE of 19.0%.

**FIGURE 2:**
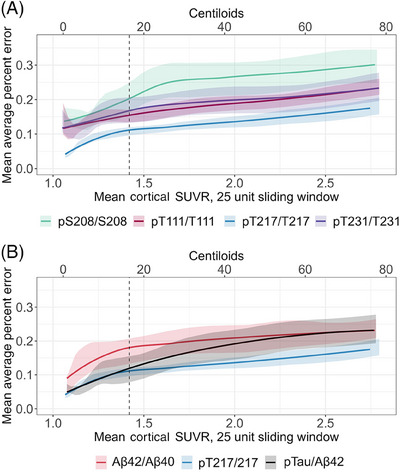
Model performance across studied range of SUVR (A and B). Models were constructed for all tau phosphorylation sites, but only models for tau phosphorylation sites with ρ > 0.75 are shown for ease of interpretation. For convenience, cortical amyloid burden as measured by PET PiB in SUVR was translated into Centiloids on a secondary axis, which is applicable regardless of PET tracer. Lower values correspond to better performance. PET, positron emission tomography; SUVR, standardized uptake value ratio.

This cohort was predominantly composed of amyloid‐negative individuals, so it was important to examine the performance of the model specifically at elevated levels of amyloid pathology. To achieve this, we evaluated model performance with MAPE using a sliding window for predicted amyloid PET Centiloid. The CSF pT217/T217 model significantly outperformed all other models across the range of cortical amyloid accumulation, with the exception of the models based on CSF pT111/T111 (Figure 2A) and Lumipulse p‐tau181/Aβ42 (Figure 2B). CSF p‐tau181/Aβ42 significantly outperformed CSF Aβ42/Aβ40 until an average amyloid value of 31 Centiloids (1.75 SUVR) was obtained, while CSF pT217/T217 significantly outperformed Aβ42/Aβ40 up to an average amyloid value of 64 Centiloids (2.48 SUVR), at which point the models performed similarly. Although only a subset of tau phosphorylation sites are shown in Figure 2 for readability, these analyses are shown for all analytes in Figure S2.

**FIGURE 3:**
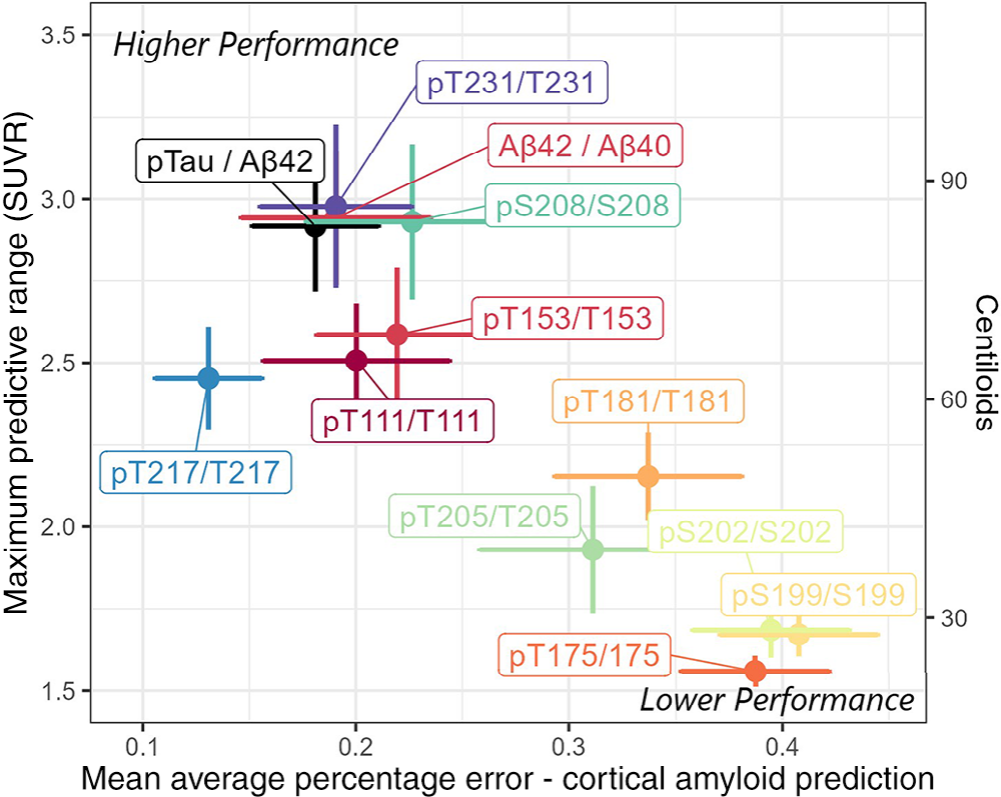
Maximum predictive range of fluid biomarkers relative to error. The upper‐left‐hand corner represents higher performance: fluid biomarkers with low error and a large predictive range. For convenience, cortical amyloid burden as measured by PET PiB in SUVR was translated into Centiloids on a secondary axis, which is applicable regardless of PET tracer. PET, positron emission tomography; SUVR, standardized uptake value ratio.

The maximum upper limit of prediction for each of the biomarkers was evaluated by asymptotic regression. CSF pT231/T231 (86.6 Centiloids, 95% CI: 75.4, 95.2) and pS208/S208 (84.4 Centiloids, 95% CI: 73.4, 95.2) had the highest predictive ranges (Figure 3, Figure S3). The horizontal asymptote of the model based on pT217/T217 was lower (62.8 Centiloids, 95% CI: 55.6, 75.8). CSF Aβ42/Aβ40 and p‐tau181/Aβ42 were similar to pT231/T231 and pS208/S208 (84.8 Centiloids, 95% CI: 75.8, 94.3 and 83.9 Centiloids, 95% CI: 74.9, 92.9, respectively) (Figure 3).

Finally, we compared the performance of the tau phosphorylation sites and CSF biomarkers at the regional level. CSF p‐tau181/Aβ42 performed well across the cortex, with MAPEs of 12.9% to 21.4% (Figure S4A). CSF pT231/T231 and pT217/T217 performed better in the gyrus rectus and lateral temporal gyrus, while CSF pT205/T205 performed best in the precuneus and prefrontal cortex (Figure S4B).

## 4 DISCUSSION

As the number of AD biomarkers rapidly expands, it is critical to compare the characteristics of these biomarkers. We predicted continuous values of mean cortical and regional amyloid burden using CSF biomarkers of tau phosphorylation to characterize the association of these biomarkers with amyloid PET over different levels of amyloid burden. These analyses demonstrate the sites at which tau is being phosphorylated as amyloid accumulates.

In addition to evaluating tau phosphorylation sites, we used CSF biomarkers obtained with clinically used Lumipulse assays. We examined the performance of CSF Aβ42/Aβ40 given its long history of use in the field of AD research^8^, ^9^, ^14^ and the CSF p‐tau181/Aβ42 ratio given recent work suggesting that p‐tau181/Aβ42 may have better concordance with amyloid PET than Aβ42/Aβ40.^10^

The biomarkers that had the highest correlation (|ρ_Spearman_| > 0.75) with mean cortical amyloid burden were CSF pT217/T217, pT111/T111, Aβ42/Aβ40, pT231/T231, and pS208/S208. However, the highest correlation with mean cortical amyloid burden did not necessarily translate into the best performance in predicting amyloid across the entire range of amyloid burden. We used ANNs, a method that was chosen for its non‐linearity, flexibility, and ability to predict multiple outputs simultaneously. Because ANNs utilize the covariance structure of the outputs when training, the ANN model predictions build upon training data generated from the entire brain rather than a single cortical summary value. This approach incorporates both spatial and temporal information into generated predictions. We observed distinct differences in the correspondence between some tau phosphorylation sites and their ability to generate regional predictions. CSF pT231/T231 and pT217/T217 predicted amyloid better in the gyrus rectus and lateral temporal gyrus, which typically develop amyloid plaques before the precuneus and prefrontal cortex (regions where pT205/T205 had better predictive ability).^32^ This corresponds to other existing evidence that indicates pT231/T231 and pT217/T217 increase before pT205/T205. These data suggest that the regional spread of amyloid^32^, ^33^, ^34^ could be directly mapped to the sequential elevations of these novel tau phosphorylation sites.^35^

In addition to considering the overall predictive performance of each biomarker, we evaluated their performance using a sliding window approach. A sliding window improves our ability to evaluate model performance across the full range of amyloid accumulation. This sliding window analysis revealed that CSF p‐tau181/Aβ42, pT217/T217, and pT111/T111 performed similarly well across the available range of values. If we had relied solely on the average MAPE estimates, we would have inferred that pT217/T217 had the strongest performance. However, its relatively lower MAPE (13% as compared to approximately 19%) was driven by its strong performance in predicting low levels of cortical amyloid.

The maximum predictive value of pT217/T217 was SUVR 2.48 (64 Centiloids), which was lower than the range for Aβ42/Aβ40 (SUVR 2.94; 84.8 Centiloids) or p‐tau181/Aβ42 (SUVR 2.92; 83.9 Centiloids), as well as many other phosphorylation sites evaluated here (Figure 3). Studies have highlighted pT217/T217 as a promising fluid biomarker with better concordance with amyloid PET than more commonly used biomarkers,^7^, ^21^ and our analysis supports this finding but introduces the important caveat that its predictive capacity plateaus at a relatively low level of amyloid accumulation. Recall this model is premised on generating region‐by‐region forecasts across the brain, including an aggregated cortical amyloid forecast. Other mechanisms independent of amyloid deposition, such as tau aggregation in the early Braak stages, are likely occurring in individuals with substantial amyloid burden. Caution should be used in interpreting results in individuals with advanced amyloidosis. Multiple pathological changes are likely occurring simultaneously.

The observed prediction plateau of CSF pT217/T217 occurs well after published thresholds for amyloid positivity.^28^ Predictive error assessed via sliding window after 20 Centiloids primarily includes model performance in participants with true amyloid signal. The gradually increasing error metric displayed in Figure 2A for pT217/T217 reflects lower performance of pT217/T217 with increasing amyloid burden. To place the predictive plateau of 65 Centiloids into a broader context, a recent drug trial for Lecanemab enrolled participants with a mean cortical amyloid burden of 77.92 Centiloids (SD 44.84 Centiloids).^36^ This suggests that while pT217/T217 could be used to accurately classify individuals as amyloid positive or negative, it may have limited ability to differentiate amyloid burden in more than half of the participants enrolled in the aforementioned trial. If fluid biomarkers are to be considered as continuous outcome measures for clinical trials, it is vital that they be assessed for correspondence with pathology across the range of pathological burden to be included in a trial.

Consistent with prior work, we observed a relatively poor predictive performance of amyloid PET burden by CSF pT205/T205. Previous work suggested that pT205/T205 had a relatively weak association with amyloid PET but a higher correspondence with tau PET^17^, ^21^, ^22^ and neurodegeneration.^37^ Analyses focused on tau phosphorylation as a continuum indicated pT205/T205 as increasing after pT217/T217 but before PET tau changes.^17^, ^35^ We hypothesized that in a cohort containing more individuals with higher amyloid and tau burden, we might observe a stronger relationship between amyloid PET and pT205/T205. This is a key limitation of our current sample as relatively few individuals had more advanced AD pathology.

An additional finding of interest was the behavior of pT111/T111. Investigation of forecasts at the individual level based on pT111/T111 revealed that, apart from a systematic overestimation of the cortical amyloid burden in a handful of amyloid‐negative individuals, pT111/T111‐driven predictions were quite good. pT111/T111 is known to play a significant role in the initial modulation of tau phosphorylation and, thus, may also play a key role in forecasting future tau aggregation.^35^ This isoform is rarely measured, but, given its strong performance here and in another recent study,^38^ as well as its established biological mechanism, it may represent an important target for future biomarker development.

When we evaluated the biomarkers on the basis of both predictive range and quality of prediction, as shown in Figure 2, we observed that CSF p‐tau181/Aβ42 and Aβ42/Aβ40 as measured with the Lumipulse assay exhibited a very high level of performance. Evidence already exists that p‐tau181/Aβ42 has a high correlation with cortical amyloid burden,^10^ and our results confirmed this observation. Taking into account this information as well as the evaluation of performance across sliding windows, we found a stronger concordance between amyloid PET and p‐tau181/Aβ42 than CSF Aβ42/Aβ40. This is notable as CSF Aβ42/Aβ40 has historically been used as a measure of amyloid, but p‐tau181/Aβ42 can be obtained with the same assay platform.

We compared the prediction of continuous amyloid PET values by CSF tau phosphorylation at different sites and traditional CSF assays. When we evaluated CSF biomarkers in terms of both their ability to predict continuous amyloid PET values as well as their maximum range of prediction, we found that pT231/T231 and pS208/S208 had characteristics similar to Lumipulse‐measured p‐tau181/Aβ42 and Aβ42/Aβ40. CSF pT217/T217 had the lowest error rate; however, its predictive range was not as large as that of the other biomarkers. In light of recent work demonstrating strong concordance of plasma pT217/T217 with CSF pT217/T217, as well as the evidence presented here, we encourage the continued development of pT217/T217 assays for determining amyloid status, although they may not predict higher levels of amyloid burden very accurately. CSF pT231/T231 and pS208/S208 may also have clinical utility given their strong performance in this study. Further, we encourage use of CSF p‐tau181/Aβ42 in lieu of Aβ42/Aβ40 given both existing evidence and the evidence presented here, which suggests it also has a stronger continuous relationship with cortical amyloid burden. Future work could also consider the combination of multiple tau phosphorylation sites, which reflect pathological change across a continuum. A measure combining multiple tau phosphorylation sites may prove to have even higher performance in predicting continuous amyloid PET burden.

## CONFLICT OF INTEREST STATEMENT

J.K.W. reports no disclosures. B.A.G. receives research support from Eli Lilly and Avid Radiopharmaceuticals. N.B. is the inventor on the following US patent applications: “Methods to detect novel tau species in CSF and use thereof to track tau neuropathology in Alzheimer's disease and other tauopathies” (PCT/US2020/046224); “CSF phosphorylated tau and amyloid beta profiles as biomarkers of tauopathies” (PCT/US2022/022906); and “Methods of diagnosing and treating based on site‐specific tau phosphorylation” (PCT/US2019/030725). N.B. may receive a royalty income based on technology licensed by Washington University to C2N Diagnostics. K.H. is an Eisai‐sponsored voluntary research associate professor at Washington University and has received a salary from Eisai. R.L.H. reports no disclosures. Y.H. reports no disclosures. S.F. reports no disclosures. T.L.S.B. has consulted on clinical trials with Biogen, Roche, Jaassen, and Eli Lilly. She receives research support from Eli Lilly and Avid Radiopharmaceuticals. Neither J.C.M. nor his family owns stock or has equity interest (outside of mutual funds or other externally directed accounts) in any pharmaceutical or biotechnology company. R.J.B. has received research funding from Avid Radiopharmaceuticals, Janssen, Roche/Genentech, Eli Lilly, Eisai, Biogen, AbbVie, Bristol Myers Squibb, and Novartis. Washington University and R.J.B. have equity ownership interest in C2N Diagnostics and receive income based on technology (stable isotope labeling kinetics, blood plasma assay, methods of diagnosing AD with phosphorylation changes, neurofilament light chain assays, and materials) licensed by Washington University to C2N Diagnostics. R.J.B. receives income from C2N Diagnostics for serving on the scientific advisory board. R.J.B. serves on the Roche Gantenerumab Steering Committee as an unpaid member. R.J.B. is an inventor on the following US patent applications: “Methods to detect novel tau species in CSF and use thereof to track tau neuropathology in Alzheimer's disease and other tauopathies” (PCT/US2020/046224); “CSF phosphorylated tau and amyloid beta profiles as biomarkers of tauopathies” (PCT/US2022/022906); and “Methods of diagnosing and treating based on site‐specific tau phosphorylation” (PCT/US2019/030725). S.E.S. has analyzed data provided by C2N Diagnostics to Washington University. She has served on advisory boards for Eisai. B.M.A. reports no disclosures. Author disclosures are available in the supporting information.

## CONSENT STATEMENT

Written informed consent was obtained from all participants.

## Supporting information

Supporting Information

Supporting Information
