# Supplementary material for: Predicting continuous amyloid PET values with CSF tau phosphorylation occupancies
Source: Alzheimers Dement. 2024 Jul 23;20(9):6365–73. doi: 10.1002/alz.14132 (PMC11497729; doi:10.1002/alz.14132)
Supplement: Supplementary file 2 — Supporting Information [file ALZ-20-6365-s002.docx]

**SUPPLEMENT**


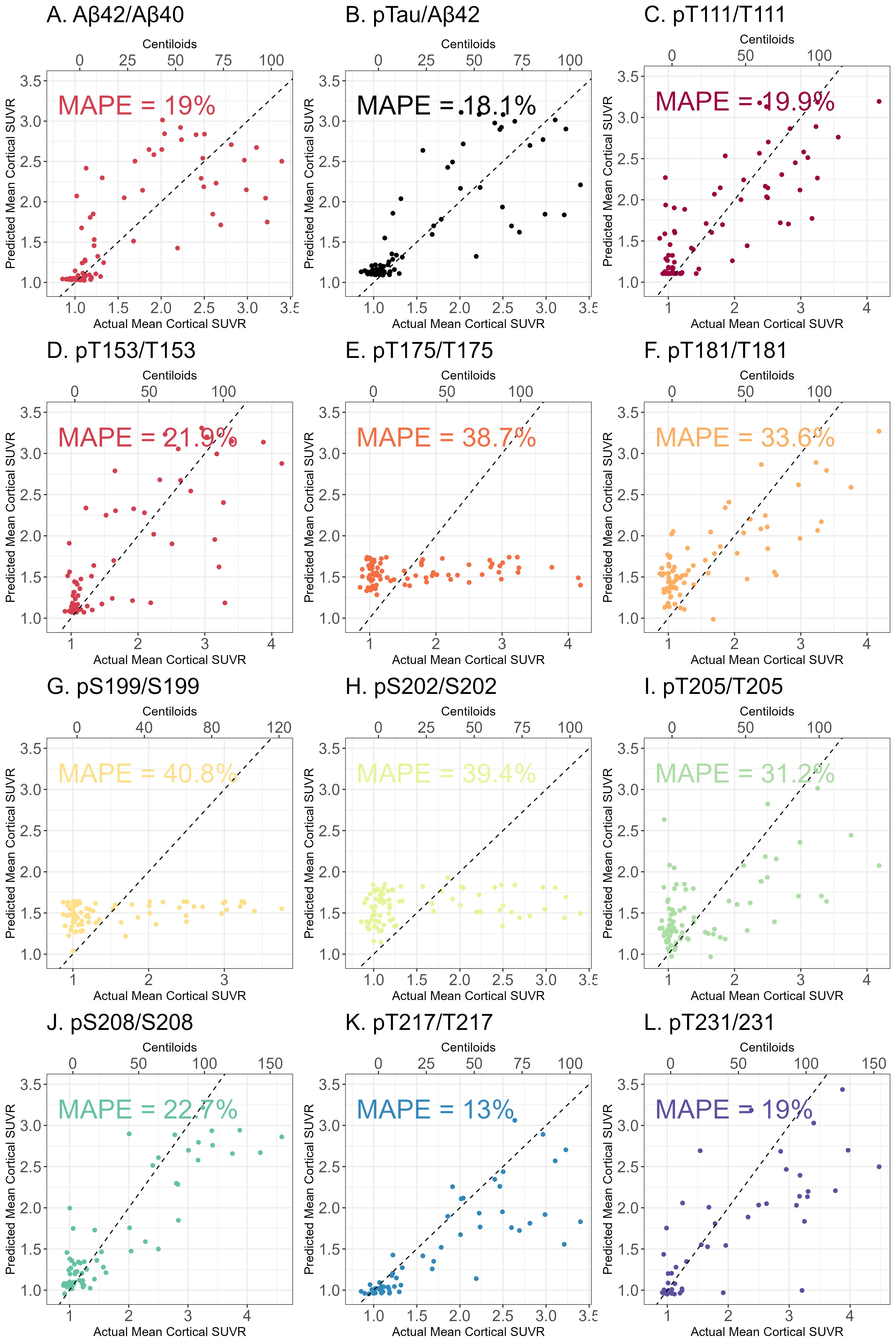


**Supplemental Figure 1. Comparison of Single Tau Phosphorylation Occupancy Based Mean Cortical SUVR Predictions to Actual Mean Cortical SUVR.** A dashed line indicates perfect correspondence between the forecasted cortical SUVR and ground truth.


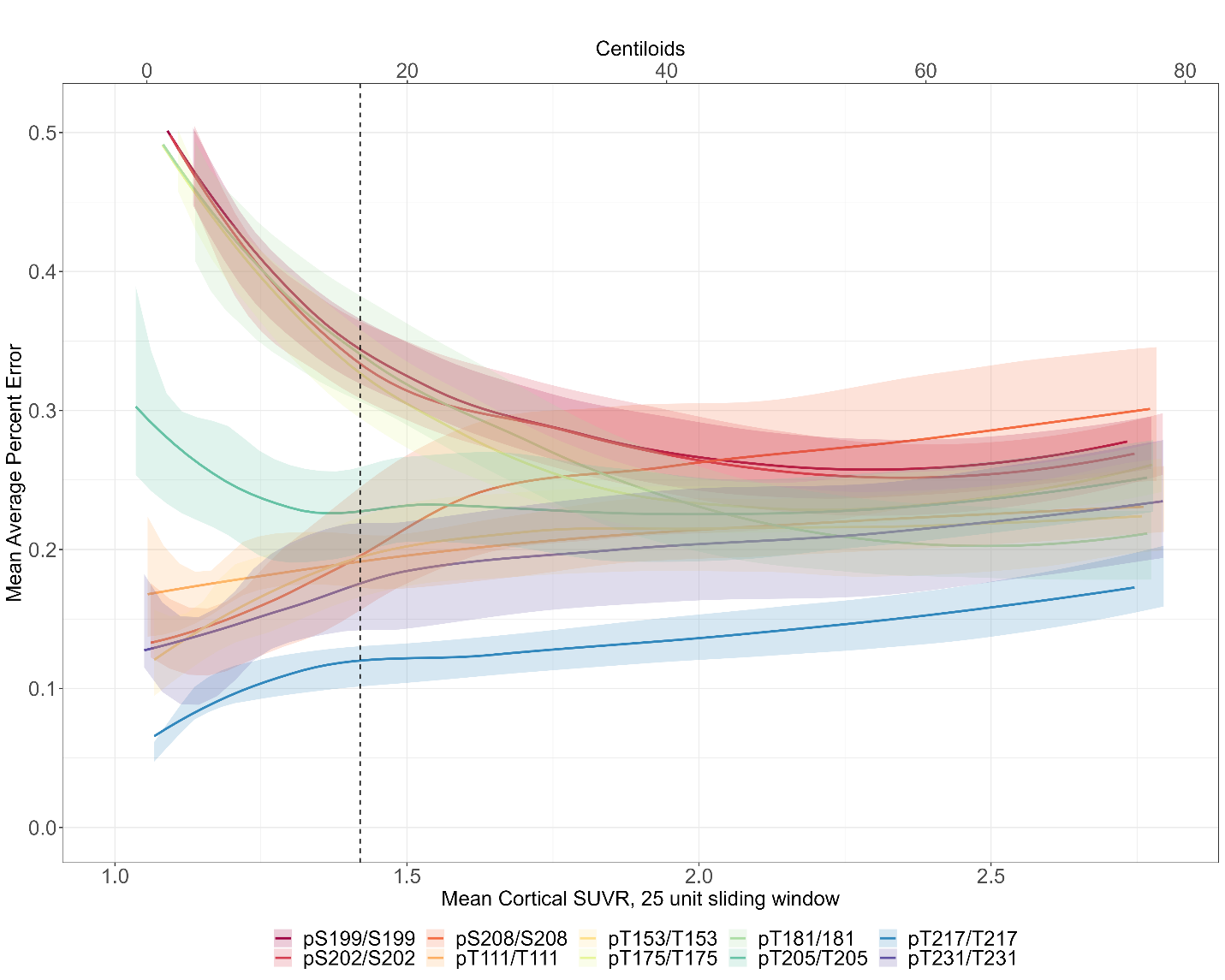


**Supplemental Figure 2. Model performance across the studied range of SUVR for all tau phosphorylation sites.** Lower values correspond to better performance.


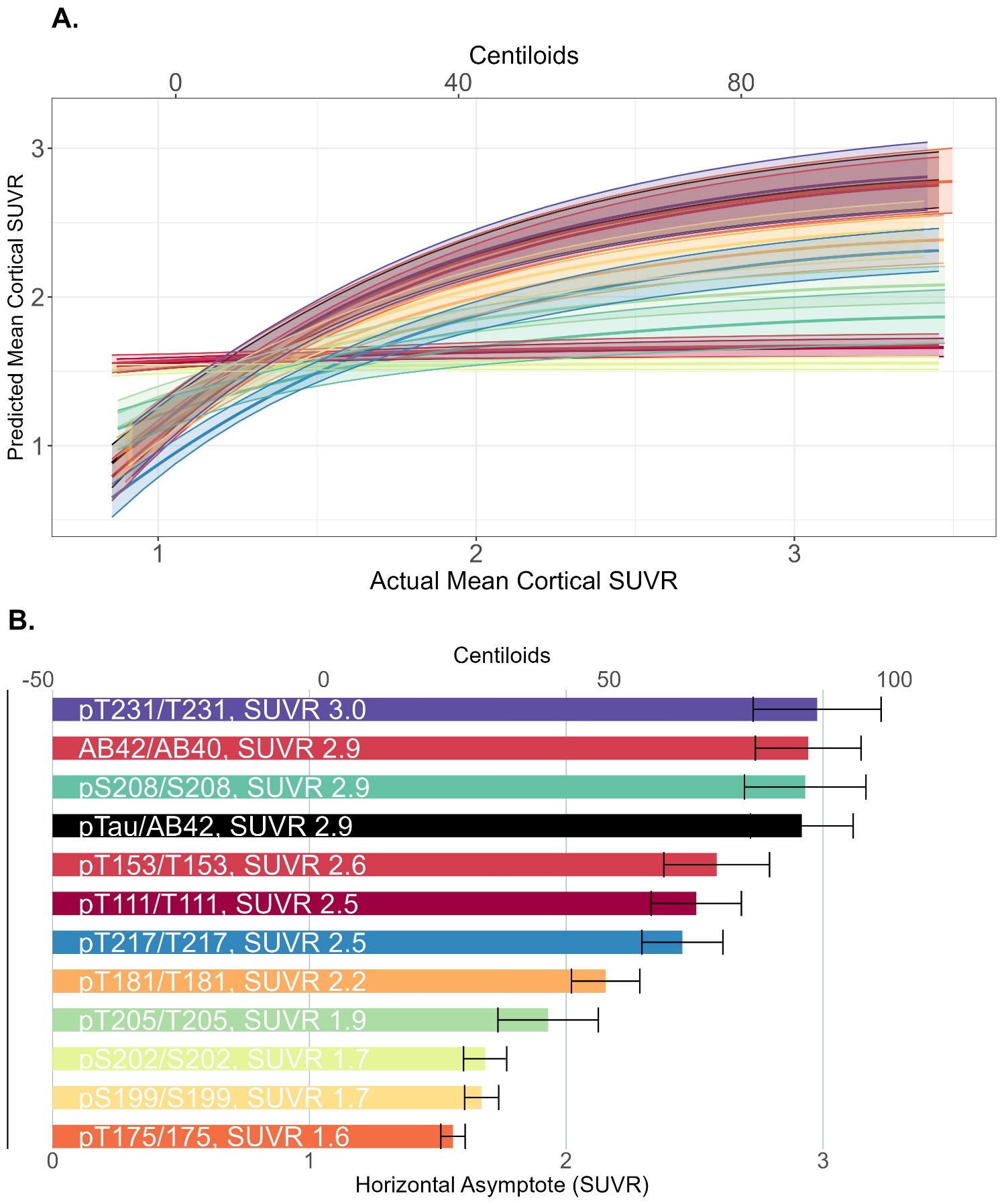


**Supplemental Figure 3. Maximum Predictive Value of Fluid Biomarkers for all tau phosphorylation sites.** Asymptotic regression of the relationship between predicted mean cortical SUVR and actual mean cortical SUVR shows that pT231/231 has the largest predictive range and pT175/T175 has effectively no predictive range (A). Calculated predictive range maxima and their associated confidence intervals are shown for all tau phosphorylation sites (B).


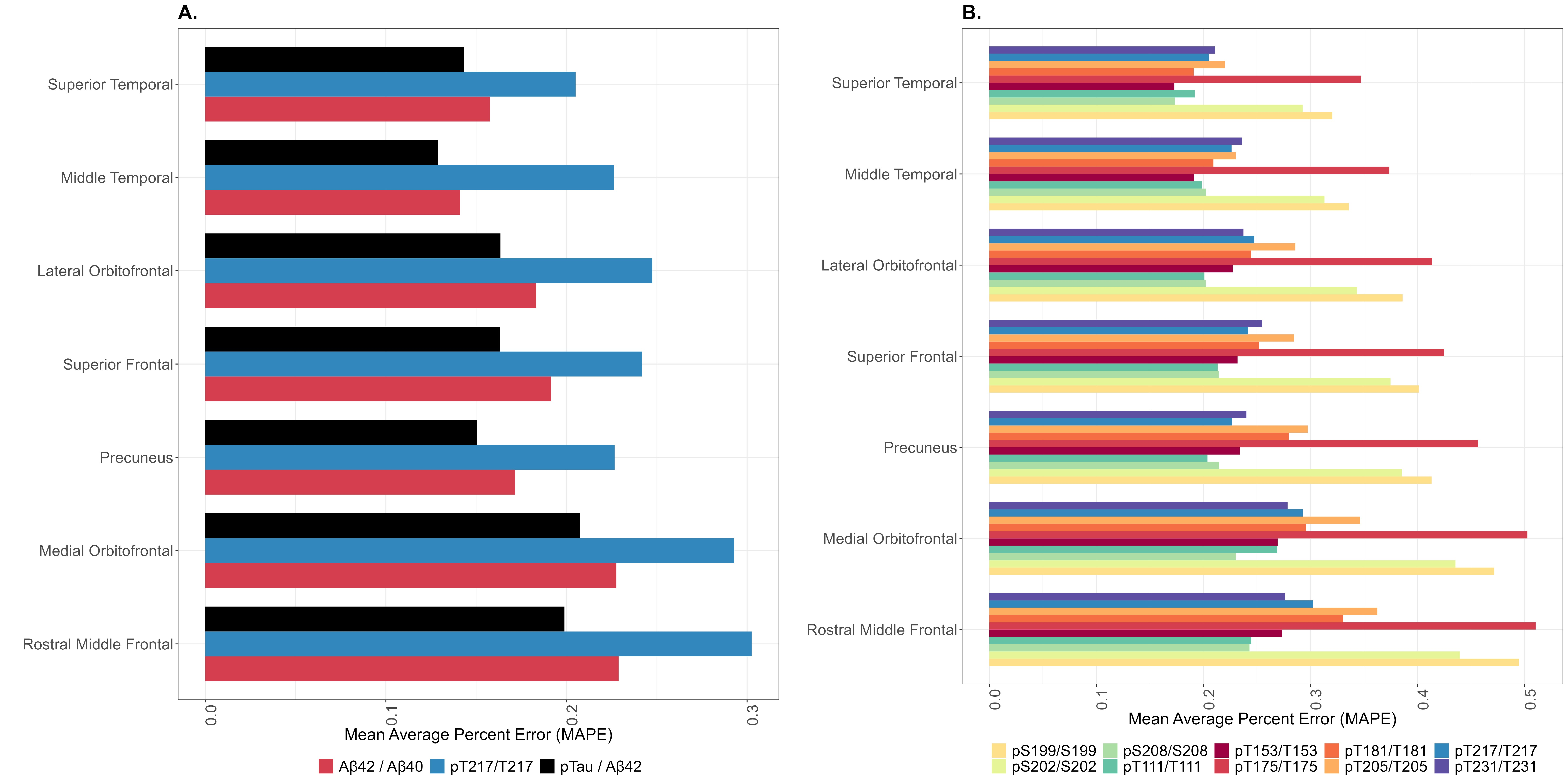


**Supplemental Figure 4. Regional level model performance.** The regions shown are cortical regions where one would expect significant amyloid accumulation in the context of preclinical Alzheimer Disease. P-tau181/Aβ42 consistently outperforms both Aβ42/Aβ40 and pT217/217 for individual regions (A). The models individually constructed with either CSF pT231/231 or pT217/T217 show the strongest performance for the gyrus rectus and lateral temporal regions (B). Models constructed individually with CSF pT205/T205 perform best in the precuneus and prefrontal cortex (B). Lower values correspond to better performance.
